# Progress in Obstetrics

**Published:** 1899-06-03

**Authors:** 


### PROGRESS IN OBSTETRICS.

**The Bacteria of the Pregnant and Puerperal Vagina and their Practical Significance.**—Whitridge Williams,[1] writing on this subject, says there always have been and are now two classes of observers whose results are absolutely contradictory. Gonner, from the examination of the vaginal secretion of 32 pregnant women, maintains that the vagina does not contain the pathogenic micro-organisms which are usually found in puerperal infection, and concluded that auto-infection was not possible, and that vaginal antiseptic douches were not necessary; while Döderlein, on the contrary, found that pyogenic organisms—both staphylococci and streptococci—were present in the lochia, and therefore concluded that auto-infection was possible, and that prophylactic vaginal douches were urgently indicated. König, agreeing with Gonner, maintains that the vagina of every pregnant woman who has not been examined is aseptic, and he also demonstrates that the vaginal secretion possesses marked bactericidal power, destroying pathogenic organisms when introduced into the vagina. This bactericidal action of the vaginal secretion König considers to be due to (1) chemical substances in the secretion, perhaps acid; (2) the antagonism between the vaginal and imported bacteria; (3) phagocytic action; (4) lack of oxygen. Later investigators, including Döderlein, Walthard, Vahle, and Kottman, have found that streptococci and other pathogenic organisms are often present. König considers that the positive results thus obtained were due to organisms being carried into the vagina from the vulva in collecting the secretion. Williams, in the present research, guarded against this by using a double tube, and in all his procedures adopted strict antiseptic measures. As the result of his work, he believes that the vaginal secretion does not contain pyogenic cocci, and therefore cannot afford the slightest support for the doctrine of auto-infection as far as they are concerned. From his researches he arrives at the following conclusions: (1) That the vaginal secretion of pregnant women does not contain the usual pyogenic cocci, having found the staphylococcus epidermis only twice in ninety-two cases, but never the streptococcus pyogenes or staphylococcus aureus or albus. (2) The discrepancy in the results of the various investigators is due to the technique by which the secretion is obtained. (3) As the vagina does not contain pyogenic cocci, auto-infection with them is impossible; and when they are found they have been introduced from without. (4) The gonococcus is occasionally found in the vaginal secretion, and during the puerperium may extend into the uterus and tubes. (5) Theoretically it is possible, but not yet demonstrated, that in very rare instances the vagina may contain bacteria, which may give rise to sapræmia and putrefactive endometritis by auto-infection. (6) Death from puerperal infection is always due to infection from without, and is usually caused by neglect of aseptic precautions on the part of the physician or nurse; (7) puerperal infection is to be avoided by limiting vaginal examinations as much as possible, and cultivating external palpation. When vaginal examinations are to be made the external genitalia should be carefully cleansed and disinfected, and the hands rendered as aseptic as for a laparotomy. Vaginal douches are not necessary, and are probably harmful.

[1] Amer. Jour. of Obstetrics, Oct., 1898, Whitridge Williams.

